# Plasmids Carrying *bla*_CMY -2/4_ in *Escherichia coli* from Poultry, Poultry Meat, and Humans Belong to a Novel IncK Subgroup Designated IncK2

**DOI:** 10.3389/fmicb.2017.00407

**Published:** 2017-03-15

**Authors:** Salome N. Seiffert, Alessandra Carattoli, Sybille Schwendener, Alexandra Collaud, Andrea Endimiani, Vincent Perreten

**Affiliations:** ^1^Institute of Veterinary Bacteriology, Vetsuisse Faculty, University of BernBern, Switzerland; ^2^Institute for Infectious Diseases, University of BernBern, Switzerland; ^3^Graduate School for Cellular and Biomedical Sciences, University of BernBern, Switzerland; ^4^Department of Infectious, Parasitic and Immune-Mediated Diseases, Istituto Superiore di SanitàRome, Italy

**Keywords:** ESBL, pAmpC, food, antibiotic resistance, animals

## Abstract

The *bla*_CMY -2/4_-carrying IncB/O/K-like plasmids of seven *Escherichia coli* strains from poultry, poultry meat and human urine samples were examined using comparative analysis of whole plasmid sequences. The incompatibility group was determined by analysis of the *inc*RNAI region and conjugation assays with strains containing the IncK and IncB/O reference plasmids. Strains were additionally characterized using MLST and MIC determination. The complete DNA sequences of all plasmids showed an average nucleotide identity of 91.3%. Plasmids were detected in *E. coli* sequence type (ST) 131, ST38, ST420, ST1431, ST1564 and belonged to a new plasmid variant (IncK2) within the IncK and IncB/O groups. Notably, one *E. coli* from poultry meat and one from human contained the same plasmid. The presence of a common recently recognized IncK2 plasmid in diverse *E. coli* from human urine isolates and poultry meat production suggests that the IncK2 plasmids originated from a common progenitor and have the capability to spread to genetically diverse *E. coli* in different reservoirs. This discovery is alarming and stresses the need of rapidly introducing strict hygiene measures throughout the food chain, limiting the spread of such plasmids in the human settings.

## Introduction

The worldwide dissemination of *Enterobacteriaceae* along with resistance plasmids carrying β-lactamase-encoding genes (*bla*) is posing an increasing threat to the public health system (Mathers et al., 2015). Poultry and poultry meat have been revealed as a large reservoir of extended-spectrum β-lactamases (ESBL) and plasmid-mediated AmpC (pAmpC) enzymes in several countries (Endimiani et al., 2012; Dierikx et al., 2013; Nilsson et al., 2014; Hansen et al., 2016; Mo et al., 2016). These resistance genes are carried by various mobile genetic elements; primarily located on plasmids which are self-replicating extrachromosomal elements (Couturier et al., 1988; Carattoli, 2009). Such plasmids not only vary in their size and compatibility to other plasmid groups, but also in their capability of transferring to various bacterial species (Carattoli, 2009, 2013).

The involvement of plasmids in the zoonotic spread of β-lactamase- and carbapenemase-encoding genes between human and animal reservoirs has already been emphasized based on the presence of similar plasmids in *Escherichia coli* from humans, poultry, and poultry meat (Leverstein-van Hall et al., 2011; Börjesson et al., 2013; Seiffert et al., 2013b; Voets et al., 2013; Guerra et al., 2014; Wang et al., 2014). However, there is a lack of firm evidence since there are only few completely sequenced and assembled plasmids to demonstrate the spread and exchange of the same plasmids in *E. coli* of human and poultry origin.

By comparing datasets from poultry, poultry meat, and clinical human isolates from Switzerland, we noticed that the pAmpC *bla*_CMY -2_ and *bla*_CMY -4_ genes were associated with plasmids belonging to the IncB/O/K groups (Endimiani et al., 2012; Seiffert et al., 2013a; Vogt et al., 2014). Other studies investigating pAmpC-producing *E. coli* have detected the nearly identical kind of plasmid in healthy humans (Baudry et al., 2009; Porres-Osante et al., 2015; Hansen et al., 2016), poultry (Dierikx et al., 2010; Hansen et al., 2016; Mo et al., 2016), poultry meat (Egervärn et al., 2014), and dogs (Hansen et al., 2016). This prompted us to perform an in-depth analysis of *bla*_CMY -2/4_-carrying IncB/O/K plasmids from *E. coli* isolated in three settings in Switzerland to determine their genetic relatedness, revealing a novel IncK-group variant, the IncK2.

## Materials and Methods

### Bacterial Strains

All strains were obtained from previous studies characterizing 3rd-generation cephalosporin-resistant *E. coli* (3GC-R-*Ec*) from poultry, poultry meat, and humans isolated at the Institute of Veterinary Bacteriology and at the Laboratory of Microbiology of the Institute of Infectious Diseases, University of Bern, Switzerland (Endimiani et al., 2012; Seiffert et al., 2013a; Vogt et al., 2014). Strains were collected between 2011 and 2014 (**Table 1**) and were selected based on the presence of *bla*_CMY -2/4_ by PCR/DNA sequencing (Seiffert et al., 2013a; Vogt et al., 2014) and on ambiguous results with the PCR-based replicon typing (PBRT) of plasmids, giving cross-reaction with either IncK and/or IncB/O PCRs (Carattoli et al., 2005).

**Table 1:** Origin, characteristics and resistance profile of the original *E. coli* strains and of the *E. coli* strains transformed with the *bla*_CMY -2/4_ containing IncK2 plasmids.

Strain	Origin (date of isolation)	Sequence type	Antibiotic resistance profile	Reference
4809.66	Human UTI, hospital (2011)	ST1431	FOX CPD CRO CTX CAZ CFZ CEF CIP NAL TMP SMZ	Seiffert et al., 2013a
5312.29	Human UTI, hospital (2014)	ST131	FOX CPD CRO CTX CAZ CFZ CEF PIP/TAZ NAL	This study
DV10	Poultry retail meat, MPP from slaughterhouse A (Apr 2013)	ST38	FOX CPD CRO CTX CAZ CFZ CEF NAL	Vogt et al., 2014
DV45	Poultry retail meat, MPP from slaughterhouse A (Apr 2013)	ST1564	FOX CPD CRO CTX CAZ CFZ CEF TMP SMZ NAL TET KAN	Vogt et al., 2014
MSA1088	Broiler cloaca, slaughterhouse A (Dec 2012)	ST38	FOX CPD CRO CTX CAZ CFZ CEF TMP TET	Endimiani et al., 2012; this study
MSA992	Broiler cloaca, slaughterhouse A (Nov 2012)	ST420	FOX CPD CRO CTX CAZ CFZ CEF PIP/TAZ NAL	Endimiani et al., 2012; this study
MSA970	Broiler cloaca, slaughterhouse B (Nov 2012)	ST420	FOX CPD CRO CTX CAZ CFZ CEF PIP/TAZ NAL	Endimiani et al., 2012; this study
SNS4809.66	DH10B transformed with *bla*_CMY -2_ plasmid p4809.66	–	FOX CPD CRO CTX CAZ CFZ CEF	This study
SNS5312.29	DH10B transformed with *bla*_CMY -2_ plasmid p5312.29	–	FOX CPD CRO CTX CAZ CFZ CEF	This study
ALC74	TOP10 transformed with *bla*_CMY -4_ plasmid pDV10	–	FOX CPD CRO CTX CAZ CFZ CEF	This study
ALC76	TOP10 transformed with *bla*_CMY -2_ plasmid pDV45	–	FOX CPD CRO CTX CAZ CFZ CEF	This study
TMSA1088	TOP10 transformed with *bla*_CMY -2_ plasmid pTMSA1088	–	FOX CPD CRO CTX CAZ CFZ CEF	This study
TMSA992	TOP10 transformed with *bla*_CMY -2_ plasmid pTMSA992	–	FOX CPD CRO CTX CAZ CFZ CEF PIP/TAZ NAL	This study
TMSA970	TOP10 transformed with *bla*_CMY -2_ plasmid pTMSA970	–	FOX CPD CRO CTX CAZ CFZ CEF PIP/TAZ	This study
TOP10	One Shot^®^ TOP10 Electrocomp^TM^ cells	–		Invitrogen, Life Technologies
DH10B	ElectroMax DH10B competent cells	–		Invitrogen, Life Technologies

*The three isolates from broiler cloacae were from three different herds. The three isolates from poultry meat were from three different lots originating from the meat packing plant (MPP) of slaughterhouse A; The three isolates from human infections were from patients hospitalized at the same hospital. CEF, cephalotin; CFZ, cefazolin; FEP, cefepime; CTX, cefotaxime; CTX-CLA, cefotaxime/clavulanic acid; FOX, cefoxitin; CPD, cefpodoxime; CAZ, ceftazidime; CRO, ceftriaxone; CHL, chloramphenicol; CIP, ciprofloxacin; CST, colistin; GEN, gentamicin; IPM, imipenem; KAN, kanamycin; MEM, meropenem; NAL, nalidixic acid; TZP, piperacillin/tazobactam; STR, streptomycin; SMZ, sulfamethoxazole; TET, tetracycline; TMP, trimethoprim*.

Plasmids were transformed by electroporation into either *E. coli* ElectroMax DH10B competent cells or into *E. coli* One Shot^®^ TOP10 Electrocomp cells (Invitrogen, Life Technologies) as previously described (Seiffert et al., 2013a; Vogt et al., 2014). The MIC of antibiotics were obtained from donors and transformants using the microdilution Sensititre panels ESB1F and EUMVS2 (Trek Diagnostics) and interpreted following the CLSI guidelines (CLSI, 2016). Sequence types (ST) were assigned following the multi-locus sequence typing (MLST) Achtman scheme^1^.

### Whole Plasmid Sequencing (WPS)

Plasmids were purified using the Genopure Plasmid Midi Kit (Roche Diagnostic) according to the manufacturer’s procedures and used for Whole-Plasmid Sequencing (WPS). WPS was performed using the 454-Junior Genome Sequencer procedure (Roche Diagnostic). The libraries of plasmid DNA were constructed using the GS FLX Titanium Rapid Library Preparation Kit.

### *De novo* Assembly of DNA Reads, Gap-Closure and Annotation

Contigs with at least a 70-fold coverage were obtained using the GS *de novo* Assembler software (Roche Diagnostics). Scaffolding was first done *in silico* by using the 454 ReadStatus output file to identify reads with overlapping adjacent contigs. The assembly and gap-closure, as well as insertion/deletion events, single nucleotide polymorphisms and shufflons were confirmed and verified by PCR followed by Sanger DNA sequencing. The annotation was done using the Artemis Version 8 (Sanger Institute) in combination with a pairwise alignment using a BLASTN and BLASTP homology search^2^.

### Phylogenetic Analysis of IncB/O/K Plasmids

For the phylogenetic analysis of the IncB/O/K-like plasmids, two fully sequenced plasmids, namely pCT belonging to IncK (GenBank acc. No. FN868832.1; Cottell et al., 2011) and p3521 belonging to IncB (GU256641; Papagiannitsis et al., 2011) were included as reference. These plasmids were chosen as they represent IncK and IncB plasmids whose complete nucleotide sequence is available in the GenBank. The phylogenetic trees and the homology matrices were constructed by UPGMA method and Jukes-Cantor correction [Multiple alignment (open gap penalty (OG): 100%, unit gap penalty (UG): 0%, gap penalty: 100%), 2000 Bootstrap trials] using Bionumerics 7.6 (Applied Maths, Kortrijk, Belgium).

The analyses were performed using the full length sequences of the IncK2 plasmids with and without shufflon region. The deleted shufflon region started at the shufflon-specific DNA recombinase (*rci*) and ended with the last shufflon gene which varies from plasmid to plasmid (position 62686–65450 in pTMSA970). In the case of the reference IncK and IncB plasmids, in addition to the shufflon region, the 3,144-bp *bla*_CTX-M-14_-carrying element was removed from the IncK reference plasmid (position 68837–71981 in FN868832) and the 26,548-bp large *bla*_ACC-4_ containing element was removed from the IncB reference plasmid (position 59569–86117 in GU256641).

### Conjugation for Incompatibility Testing

Mating experiments were performed crossing the transformant strain containing plasmid p5312.29 (strain SNS5312.29) or pTMSA970 (strain TMSA970) with reference plasmids Rhh72 (IncB/O), and R387 (IncK; provided by the Istituto Superiore di Sanità, Rome, Italy) which are used for incompatibility testing (Couturier et al., 1988). Conjugations were performed on Luria-Bertani (LB) agar solid media streaking two crossing lines, one for each mating strain, and incubating the mating plates overnight at 37°C.

Donor and transconjugant colonies were counted after plating 10-fold serial dilutions on LB plates containing 50 μg/ml of ampicillin (as resistance marker for p5312.29 and pTMSA970), 50 μg/ml kanamycin (as resistance marker for Rhh72), 25 μg/ml chloramphenicol (as resistance marker for R387), 50 μg/ml of ampicillin plus 50 μg/ml kanamycin, and 50 μg/ml of ampicillin plus 25 μg/ml chloramphenicol.

Stability of the plasmids within the conjugant bacterial cells was determined as follows: three independent colonies resulting from each mating and exhibiting the double resistance (ampicillin/kanamycin, or ampicillin/chloramphenicol) were grown 24 h at 37°C in 50 ml of LB without antibiotics. Ten-fold dilutions of the conjugant cultures were plated on LB plates containing either ampicillin, kanamycin or chloramphenicol alone, and on plates containing both ampicillin and kanamycin, or both ampicillin and chloramphenicol. Colonies were counted after an overnight incubation at 37°C. Stability was determined as the ratio between the number of ampicillin-resistant colonies and the number of ampicillin/kanamycin-resistant colonies for the p5312.29 × Rhh72 and pTMSA970 × Rhh72 transconjugants, and between the number of ampicillin-resistant colonies and the number of ampicillin/chloramphenicol-resistant colonies for the p5312.29 × R387 and pTMSA970 × R387 transconjugants, respectively.

### Nucleotide Sequence Accession Numbers

The complete nucleotide sequence of the seven IncK2 plasmids have been deposited into the GenBank database under accession numbers KR905385 (p5312.29), KR905389 (p4809.66), KR905387 (pTMSA992), KR905388 (pTMSA970), KR905386 (pTMSA1088), KR905384 (pDV45), and KR905390 (pDV10).

## Results and Discussion

### Plasmid Sequencing

Whole plasmid sequencing of seven *bla*_CMY -2/4_-containing plasmids [poultry cloacae: pTMSA970, pTMSA992, pTMSA1088; poultry meat: pDV10, pDV45; human urine isolates: p4809.66, p5312.29] revealed the presence of highly related plasmids in both genetically related and diverse *E. coli* from poultry (ST38, ST420), poultry meat (ST38, ST1564) and human clinical samples (ST131, ST1431).

**FIGURE 1:**
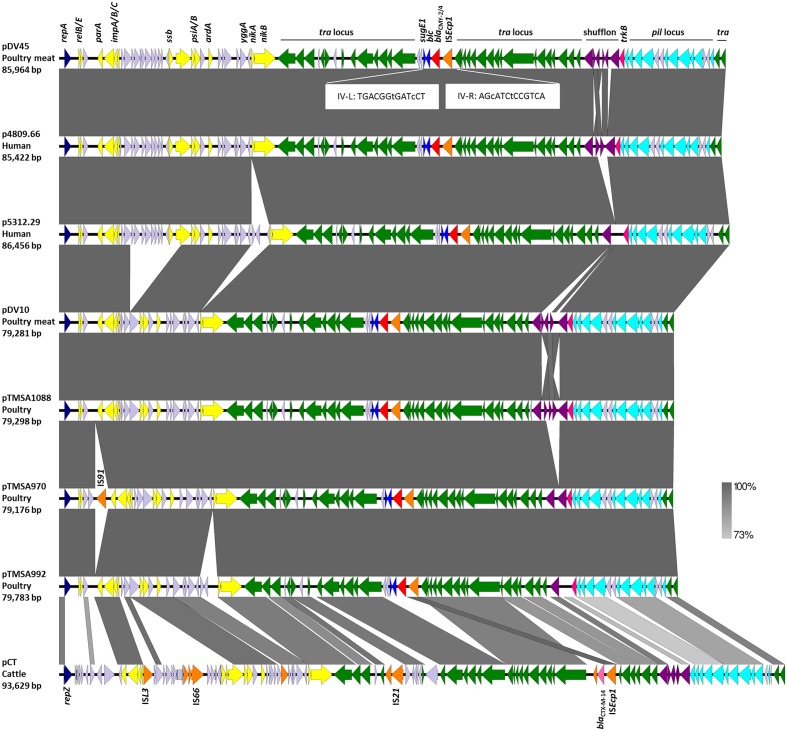
**Structural features of seven fully sequenced IncK2 plasmids encoding CMY-2/4 β-lactamases and of the IncK plasmid pCT**. The open reading frames are illustrated by arrows pointing in direction of their respective orientation. The figure was generated with EasyFig 2.1 (Sullivan et al., 2011). Imperfect inverted repeats (IV-R and IV-L) are shown in white boxes. The color code is as follows: The *tra* and *trb* transfer loci are in green, antibiotic resistance genes in red (*bla*_CMY -2/4_) and pink (*bla*_CMY -2/4_), insertion sequence element (IS) family in orange, replicase genes in dark blue, partitioning genes and toxin/anti-toxin and other stabilization systems in yellow, shufflon-associated genes in violet, pilus genes in pale blue, *sugE1* and *blc* genes in blue, and genes with unknown function are in lavender. Important genes and loci are indicated above the first plasmid sequence. Plasmid sequences of pTMSA970 and pCT carry additional IS elements which are indicated in the figure. The black lines above the figure indicate the different loci and the shufflon-region.

Plasmids mainly differed from each other within the highly variable shufflon region, and a region situated between *impC* and *nikA* (**Figure 1**). A major deletion of approximately 6 kb, that starts after *yfbA* and ends before *psiB*, distinguished plasmids pDV10, pTMSA1088, pTMSA970, pTMSA992 from the larger plasmids pDV45, p4809.66, and p5312.29 (**Figure 1**).

Another fragment of 2.3 kb situated between *yggA* and *nikA* distinguished plasmids p5312.29 and pTMSA992 from the other plasmids (**Figure 1**). Plasmid pTMSA970 also had an extra insertion of a previously unreported IS-element which is closely related to IS*Sbo1* (GenBank acc. no. CP001062) and belongs to the IS*91* family. Integrations or losses of these regions might have occurred as single events during the transfer of the plasmids from one bacterial host to the other rather than during a longer evolutionary process. Outside of these regions, the plasmid sequences were virtually identical, only differing by a few SNPs, which have all been confirmed by Sanger sequencing.

Of note, except for the variable shufflon regions, plasmid pDV45 from *E. coli* from poultry meat was identical to plasmid p4809.66 from human *E. coli*, and plasmid pDV10 *E. coli* from poultry meat was identical to plasmid pTMSA1088 from poultry (**Figure 1**), which strongly suggests transfer of plasmids between genetically diverse *E. coli* along the food chain.

The variable region between *impC* and *nikA* also harbors, along with many genes encoding for proteins with unknown function the single-stranded DNA-binding (Ssb) protein. One of the main functions of the Ssb protein is binding of ssDNA and subsequent protection from degradation by nucleases (Meyer and Laine, 1990). The direct consequence of the loss of Ssb for the IncB/O/K-group plasmids is difficult to determine and needs further investigations.

The shufflon is a biological switch that controls the C-terminal end of the PilV proteins via site-specific recombination. This mechanism has been described in R64 as well as in pCT where the shufflon determines the recipient specificity in liquid mating despite being part of a minor component of the thin pilus (Komano, 1999; Cottell et al., 2014). The shufflon of R64 consists of four invertible DNA fragments embedded with seven 19-bp repeat sequences. These 19-bp repeats can be subdivided into a 7-bp core-site region where the DNA crossover occurs and the highly conserved 12-bp right arm region (Komano, 1999; Brouwer et al., 2015).

The shufflon of the seven plasmids is highly diverse and contains between two and eight recombination sites. Four of the 19-bp repeat sequences found in R64 were also identified in the plasmids. Interestingly, the 7-bp core-site region is identical to the ones identified in the IncI1/I2 plasmids. The terminology of 19-bp repeat sequences follows the designation of the repeats in R64 (Komano, 1999) (i.e., repeat-e: *GTGCCAA*TCCGGTtcgTGG; repeat-f: *GTGCCAA*TCCGGTatcTGG; repeat-g: *GTGCCAA*TCCGGTGtgtGG; repeat-h: *GTGCCAA*TCCGGTttgTGG; letters in *italic* represent the core-site, small letters for the variable bases). The three plasmids pTMSA970, pTMSA992, and p5312.29 have only two repeat-e sequences, followed by p4809.66 with four repeat sequences in the following order: e-e-e-g. The most complex shufflons with eight repeat sequences each were detected in pDV10 (g-g-e-e-e-e-h-f), pDV45 (e-e-e-e-g-g-h-f), and pTMSA1088 (e-e-h-f-e-e-g-g). Although, no apparent correlation between the numbers of recombination sites could be detected (neither with the size of the plasmid nor with their origin), the observation of such great diversity may have an impact on the mating preferences of the various plasmids.

### Incompatibility Assays

**FIGURE 2:**
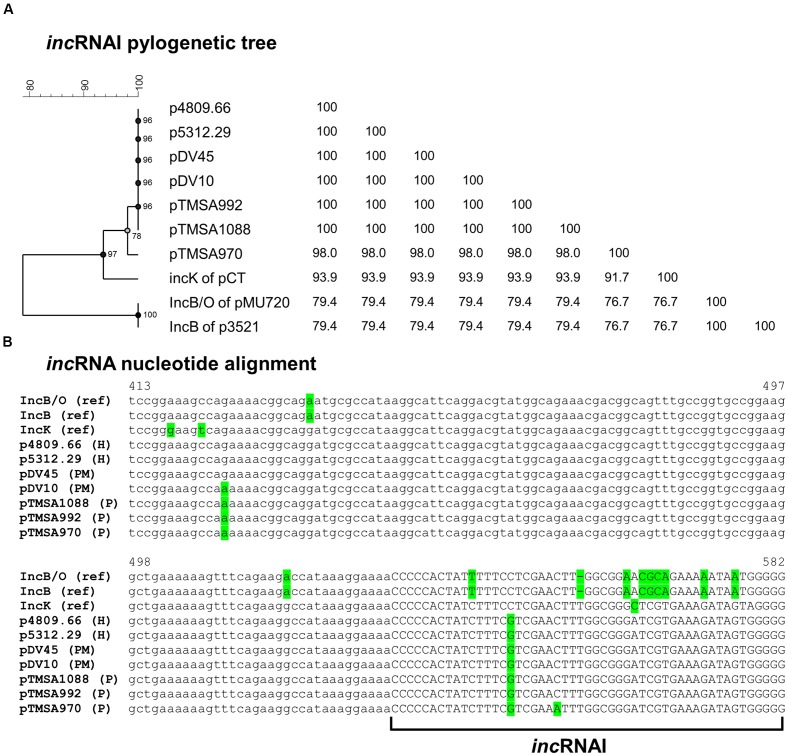
**(A)** Phylogenetic tree and **(B)** sequence alignment of the *inc*RNAI-II of the IncB/O/K-group plasmids including references. The sequences were extracted from the IncK2 plasmids as well as from the reference plasmids of IncB and IncK. The IncB/O *inc*RNAI-II reference sequence was derived from the paper of Couturier et al. (1988). Due to the absence of the complete nucleotide sequence of the IncB/O plasmid, pMU720 was not included into **Figure 3**. The phylogenetic tree was constructed by UPGMA method and Jukes-Cantor correction [Multiple alignment (open gap penalty (OG): 100%, unit gap penalty (UG): 0%, gap penalty: 100%), 2000 Bootstraps] using Bionumerics 7.6 (Applied Maths, Kortrijk, Belgium). H, human urine sample; P, poultry; PM, poultry meat.

Sequence analysis of the region upstream of the *repA* including the *inc*RNAI revealed that the DNA sequences of the seven plasmids diverged from the previously reported *inc*RNAI of the IncK, IncB, and IncB/O plasmids (**Figure 2**).

This region before the *repA* is highly conserved among the specific plasmid Inc groups and is crucial for the incompatibility behavior among plasmids of the same group (Couturier et al., 1988; Praszkier et al., 1991). The key element for this behavior is the *inc*RNAI that forms a stem and loop structure and negatively regulates the transcription of *repA*. Consequently, if two plasmids of the same or similar Inc group(s) reside in the same bacterial cell the *inc*RNAIs hybridize with each other and prohibit translation (Couturier et al., 1988). Additionally, a phylogenetic tree constructed using only the *inc*RNAI sequences containing the stem-and loop structure strengthened the previous observations that the seven plasmids are highly similar to each other, and are more closely related to the IncK group than the IncB/O groups (**Figure 2**).

The divergent *inc*RNAI sequences suggested that these plasmids could be compatible with the IncK and IncB/O reference plasmids. Therefore, incompatibility assays were performed crossing SNS5312.29 and TMSA970 strains with both IncB/O Rhh72 and IncK R387 reference strains, respectively. Transconjugants were obtained from all mating experiments, but at different frequency. Both SNS5312.29 and TMSA970 showed higher frequencies of conjugation when crossed with IncB/O (1 × 10^-5^ transconjugant/donor cell) than when crossed with the IncK (1 × 10^-7^ transconjugant/donor cell) reference plasmid. This suggests that the plasmids under study are more closely related to IncK than to IncB/O. Furthermore, IncB transconjugants demonstrated higher stability (>90%) when grown in liquid medium without antibiotics then the IncK transconjugants (>50%). Based on both conjugation results and *inc*RNAI sequence analysis, the plasmids were assigned to a novel variant of the IncK group designated as IncK2.

### Phylogenetic Analysis of the IncK2 Plasmids

The 4,198-bp long *bla*_CMY -2/4_-like-carrying mobile element (IS*Ecp1*-*bla*_CMY -2/4_-*blc*-*sugE1*) which is flanked by two 13-bp imperfect inverted repeats (IV-R and IV-L) was integrated between the *traT* and *traU* in all seven plasmids. Integration of IS*Ecp1*-*bla*_CMY -2/4_-*blc*-*sugE1* at the same site inside the *tra* locus is a solid indication that all the IncK2 plasmids analyzed have a common ancestor (**Figure 1**).

The same structure (IS*Ecp1*-*bla*_CMY -2/4_-*blc*) has already been reported in IncK plasmid from *E. coli* of human origin in Spain and from *E. coli* from chicken meat in Norway (Porres-Osante et al., 2015; Mo et al., 2016), as well as in IncA/C plasmids from *Salmonella enterica* subsp. *enterica* serotype Newport in the USA (Cao et al., 2015). Notably, the only *bla*_CMY -4_-carrying plasmid pDV10 found has also developed from the same progenitor as the other plasmids and was subjected to subsequent evolution via a Trp221Arg substitution in CMY-2.

**FIGURE 3:**
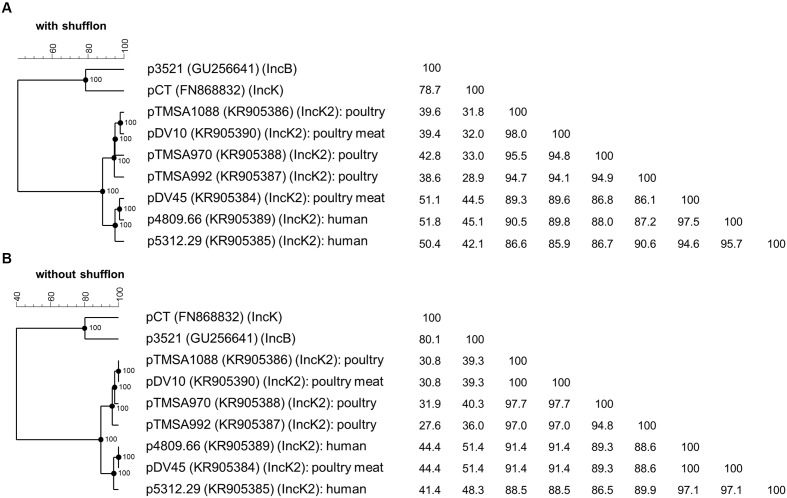
**Phylogenetic analysis and identity matrix of the IncK2 group of plasmids of *E. coli* from poultry, poultry meat, and human**. The analysis includes the plasmids of the novel IncK2 group with **(A)** and without shufflon **(B)** as well as core sequences of the reference plasmids pCT of the IncK group and p3521 of the IncB group; the reference plasmid pMU720 of the IncB/O group was not included in the tree due the absence of complete plasmid sequence. GenBank accession numbers are indicated in parentheses. The phylogenetic trees and the homology matrices were obtained by UPGMA method and Jukes-Cantor correction [Multiple alignment (open gap penalty (OG): 100%, unit gap penalty (UG): 0%, gap penalty: 100%), 2000 Bootstraps] using Bionumerics 7.6 (Applied Maths, Kortrijk, Belgium).

The phylogenetic analysis of the IncK2 plasmids supports the hypothesis of a common ancestor. All the IncK2 plasmids share a nucleotide identity ranging from 86.1 to 98.0% which increases to 86.5 to 100% once the shufflon region has been removed. This similarity is remarkable given that the percentage of identity for the two reference plasmids IncK and IncB is maximally 78.7% and without the shufflon region 80.1% (**Figure 3**). The IncK2 plasmids differed from the IncK plasmid pCT not only by their incompatibility and replication gene, but also in their structure and base composition (**Figure 1**).

Taken together, these results lead to the conclusion that the IncK2 plasmids are an overall highly homogenous cluster although they are distributed in genetically diverse *E. coli* isolated from different sources and timeframes. The phylogenetic analysis points toward a common ancestor that has subsequently spread between the various reservoirs. Indeed, the most striking characteristic of the IncK2 group is its occurrence in different reservoirs (including poultry, poultry-meat, and humans isolates) from which they were isolated. There is strong evidence that all IncK2 plasmids originated from a common source due to: (i) the identical integration site of the *bla*_CMY_-carrying mobile element; (ii) the overall 91.3% identity among all the plasmids.

Publications from Denmark, Japan, the Netherlands, Norway, Spain, and Sweden investigating pAmpC-producing *E. coli* isolates from healthy and hospitalized humans as well as strains from poultry and poultry-meat have reported a high prevalence of *bla*_CMY -2_ in both reservoirs (Dierikx et al., 2010; Börjesson et al., 2013; Voets et al., 2013; de Been et al., 2014; Egervärn et al., 2014; Huijbers et al., 2015; Porres-Osante et al., 2015; Hansen et al., 2016; Mo et al., 2016). These publications were all using the PBRT kit for characterization of their *bla*_CMY -2_-carrying plasmids, whereby a substantial part of them was either identified as IncK plasmids or as IncB/O plasmids.

Based on the present study, it has to be noted that the IncK2 plasmid-carrying strains may have been falsely identified as positive for the IncK or IncB/O plasmid groups using the PBRT. Indeed, sequence alignment of our IncK2 plasmids with DNA sequences from the GenBank revealed that such plasmids have already been identified in *E. coli* from poultry origin in different countries. One plasmid (GenBank acc. no. JXMX01000007.1) isolated from *E. coli* strain 53C from retail chicken meat in the Netherlands (de Been et al., 2014) was almost identical to pDV45 (also from chicken meat) with 98.2% nucleotide identity and 100% without considering the shufflon. Another plasmid, pNVI1292, from *E. coli* strain 2012-01-1292 (GenBank acc. no. KU312044) from retail chicken meat in Norway (Mo et al., 2016) was nearly identical to pDV10 (also from poultry meat) and to pTMSA1088 (poultry) with 98.7 and 97.8% nucleotide identity, respectively, and 100% without considering the shufflon. Of note, strain 2012-01-1292 also belonged to ST38 like both strains harboring pDV10 and to pTMSA1088 indicating that specific clones may also contribute to the spread of the IncK2 plasmids.

The novel IncK2 group seems to be an emerging epidemic plasmid with a potential for dissemination in *E. coli* from different reservoirs including human and poultry. To our knowledge, this is the first time that a highly conserved plasmid family is described in isolates from human clinical samples, poultry, and poultry meat all coming from the same geographic region. However, further epidemiological studies are necessary to assess the scope of the spread of this plasmid in both human and animals. Nevertheless, the presence of a common plasmid in three different settings is alarming and should stimulate rapid introduction of adequate measures in the meat production chain to limit the spread of drug-resistant plasmids.

## Author Contributions

Conception and design (VP, AE, SNS, AC); acquisition of data (VP, AE, SNS, AC, ACo, SS); analysis and interpretation of data (VP, AE, SNS, AC); drafting the work (VP, AE, SNS, AC); critical revison for important intellectual content (all authors); final approval of the version to be published (all authors); agreement to be accountable for all aspects of the work in ensuring that questions related to the accuracy or integrity of any part of the work are appropriately investigated and resolved (all authors).

## Conflict of Interest Statement

The authors declare that the research was conducted in the absence of any commercial or financial relationships that could be construed as a potential conflict of interest.

## Footnotes

1 http://mlst.warwick.ac.uk/mlst/dbs/Ecoli

2 http://blast.ncbi.nlm.nih.gov/Blast.cgi
